# A Review of Antioxidant Peptides Derived from Meat Muscle and By-Products

**DOI:** 10.3390/antiox5030032

**Published:** 2016-09-20

**Authors:** Rui Liu, Lujuan Xing, Qingquan Fu, Guang-hong Zhou, Wan-gang Zhang

**Affiliations:** 1 Key Laboratory of Meat Processing and Quality Control, Ministry of Education China, Jiangsu Collaborative Innovation Center of Meat Production and Processing, Quality and Safety Control, College of Food Science and Technology, Nanjing Agricultural University, Nanjing 210095, China; 2012108070@njau.edu.cn (R.L.); 2013108052@njau.edu.cn (L.X.); fuqingquan@126.com (Q.F.); ghzhou@njau.edu.cn (G.Z.); 2 School of Biochemical and Environmental Engineering, Nanjing Xiaozhuang University, Nanjing 211171, China

**Keywords:** antioxidant peptides, protein hydrolysis, solvent extraction, antioxidant capacity, purification and identification

## Abstract

Antioxidant peptides are gradually being accepted as food ingredients, supplemented in functional food and nutraceuticals, to positively regulate oxidative stress in the human body against lipid and protein oxidation. Meat muscle and meat by-products are rich sources of proteins and can be regarded as good materials for the production of bioactive peptides by use of enzymatic hydrolysis or direct solvent extraction. In recent years, there has been a growing number of studies conducted to characterize antioxidant peptides or hydrolysates derived from meat muscle and by-products as well as processed meat products, including dry-cured hams. Antioxidant peptides obtained from animal sources could exert not only nutritional value but also bioavailability to benefit human health. This paper reviews the antioxidant peptides or protein hydrolysates identified in muscle protein and by-products. We focus on the procedure for the generation of peptides with antioxidant capacity including the acquisition of crude peptides, the assessment of antioxidant activity, and the purification and identification of the active fraction. It remains critical to perform validation experiments with a cell model, animal model or clinical trial to eliminate safety concerns before final application in the food system. In addition, some of the common characteristics on structure-activity relationship are also reviewed based on the identified antioxidant peptides.

## 1. Introduction

Reactive oxygen species (ROS) including •OH, O_2_•^−^, HO_2_•, and ROO•, and reactive nitrogen species (RNS) including NO• and •ONOO, are well-known as free radicals produced by endogenous oxidation-reduction (REDOX) reactions in eukaryotes [1]. At the physiological level of ROS/RNS, they are useful as important signaling molecules to facilitate an array of biological processes, such as gene expression, cell proliferation, angiogenesis, programmed cell death, and senescence [2,3]. Oxidative and nitrosative stresses occur when accumulated ROS/RNS surpass the threshold of the body due to the large range of exogenous factors including irradiation (X-rays, *ã*-rays, ultraviolet-rays), chemical reagents (metal ions, HONOO, ozone H_2_O_2_, HOCl, and HOBr), environmental pollutants, drugs and their metabolites, xenobiotics, and smoking [4]. Superfluous ROS and RNS can cause damage to the biomacromolecule of the soma such as DNA, sugars, proteins and lipids, involved in various diseases including hypertension, cancer, diabetes, neurodegenerative disorders, heart disease, stroke, and aging [5,6].

In the human body, the cell employs a panel of enzymatic and non-enzymatic defense mechanisms to counteract oxidative and nitrosative stresses. The endogenous enzymes of superoxide dismutase (SOD), catalase, peroxidases, and thioredoxin are responsible for scavenging free radicals and natural antioxidants and also play a vital role in oxidative defense systems such as ascorbate, glutathione, tocopherol, flavonoids, alkaloids, and carotenoids [7,8,9]. Once the redox balance is disrupted causing health risks, it is necessary to reinforce the intake of antioxidant groups from food sources. This is because the amino acids, peptides and proteins derived from the food system could also function as antioxidants to protect cells and organisms from oxidative damage. For decades, natural antioxidants like vitamin C, herbal extracts and artificial antioxidants like butyl hydroxy anisd (BHA) have been commercialized [10]. Though the synthetic antioxidants of BHA and butylated hydroxytoluene (BHT) have strong antioxidant ability as food additives to prevent deterioration, they are also reported to have adverse effects on human enzyme systems and DNA [11,12]. Therefore, a large number of studies has been conducted to explore and develop safe and natural antioxidant compounds as well as antioxidant peptides [13,14].

Meat protein is thought to be a good source from which to obtain antioxidant peptides, because meat proteins contain essential amino acids in high availability that are not usually found in plant proteins, such as methylhistidine and hydroxymethyllysine. The well-known antioxidant peptides of carnosine and anserine exist endogenously in muscle tissue, acting as free radical scavengers and metal ion chelators [15]. In addition, the large abundance of meat by-products like skins, bones, meat trimmings, blood, and horns are not of high-value use in the meat industry [16], giving utilization potential for antioxidant hydrolysates and peptides. Therefore, this paper reviews the current reports about antioxidant peptides and hydrolysates, isolated from sources of meat proteins, and gives a general procedure for purification and identification of the bioactive peptides as well as the characteristics of the antioxidant peptides.

## 2. General Procedures for the Production of Antioxidant Peptides

### 2.1. Preparation of Antioxidant Fractions

The antioxidant hydrolysate and peptides generated from meat proteins are summarized in Table 1. According to this, the material of meat proteins for isolating antioxidant peptides can be divided into the following categories: raw meat, meat by-products, and meat products. Proteins of raw meat can be broadly classified as sarcoplasmic proteins, myofibrillar proteins, and salt insoluble proteins, such as collagen and elastin [17]. Collagen is the major component of meat by-products of skin and bones and is also constituted in horns. These proteins can be cleaved by exogenous protease to generate peptides, some of which have antioxidant capacity. The sources of meat processing products are dry-cured hams including Spanish dry-cured ham [18,19,20], Chinese Jinhua ham [21], and Chinese Xuanwei ham [22]. Small peptides are formed by endoenzymes to hydrolyze the hams proteins (porcine skeletal muscle) during the ripening period [23]. Due to the different routes of generating antioxidant peptides, the crude antioxidant peptides could be acquired directly by extraction and indirectly by enzymatic hydrolysis. 

#### 2.1.1. Solvent Extraction

Raw meat of chicken could exert antioxidant capacity by direct extraction [24]. The hydrophilic fraction of chicken breast and thigh meat was extracted by distilled water by homogenization and centrifugation while extraction of the lipophilic fraction was conducted by methanol:chloroform solution (2:1) followed by homogenization and filtration described by Folch et al. [25]. The Trolox equivalent antioxidant capacity of the hydrophilic fraction was 40-fold higher than that of the lipophilic suggesting that the major fraction of the antioxidant peptides was water-soluble. The antioxidant fraction derived from fermented meat sauce (FMS) was obtained by direct centrifugation of fermented mash. FMS exhibited 0.55 µmol Trolox equal/mL of DPPH radical scavenging activity and 61.2% of hydroxyl radical scavenging ability [26].

The extraction buffer of Chinese Jinhua ham and Spanish dry-cured ham was 0.01 N HCl while the Xuanwei ham was extracted with 0.2 mM phosphate buffer at pH 7.2 (Table 1). Crude extracted peptides using 0.01 N HCl from Jinhua ham showed DPPH scavenging ability of 77.39%, and that using 0.2 mM phosphate buffer from Spanish dry-cured ham of 35.34% at 1 mg/mL. However, it was not comparable because of variation in materials and antioxidant measuring systems between the different studies. Thus, the effects of different extraction methods (0.01 N HCl vs. 0.2 mM phosphate buffer, pH 7.2) on antioxidant activity of crude peptides from Jinhua ham were investigated [27]. The crude peptides extracted by 0.2 mM phosphate buffer showed higher total antioxidant capacity than that of 0.01 N HCl while no significant differences of DPPH radical scavenging capacity and superoxide anion radical scavenging activities were observed in crude peptides between the two extraction methods. Different extraction buffers may result in variations of antioxidant ability for crude extracts, because 0.01 N HCl can supply strong acid conditions which may irreversibly damage the antioxidant activity of the crude peptide while phosphate buffer can contribute to balancing osmotic pressure, ionic strength, and stabilizing pH in the extraction system [27].

#### 2.1.2. Enzymatic Hydrolysis

Enzymatic hydrolysis of target proteins is the most commonly used method for producing antioxidant peptides and hydrolysate. The mechanism is that hydrolysis releases the more active acid R groups which are inactive in parental proteins and promote hydrophobicity due to unfolding protein chains [28]. Table 1 lists different enzymes and/or processes that have been used in making protein hydrolysate and peptides. Pepsin and papain are preferred in most cases such as hydrolysis of duck skin by-products [29], porcine myofibrillar proteins [30], chicken breast protein [31], bovine brisket sarcoplasmic protein [32], venison muscle [33], porcine muscle [34], porcine blood [35] and pork hams [36]. Industrial food-grade proteinase including Alcalase, Flavourzyme, and Protamex are used for the production of antioxidant peptides in duck breast meat [37] and porcine blood plasma [38,39,40]. Multienzymes are also applied for sequential hydrolysis in gelatin from duck skin employing collagenase and pepsin [41], bovine skin gelatin employing Alcalase, pronase E amd collagenase [42], porcine skin collagen using a cocktail mixture of three enzymes from pancreas, Streptomyces, and *Bacillus polymyxa* [43]. 

Different enzymes have specificity for cleavage of individual patterns of the peptide bond [44]. Therefore, the type of proteases is the main factor for the size, amount, composition, and amino acid sequence of the peptides, and finally affects the antioxidant activity of the hydrolysate. It is crucial to guarantee the conditions of the catalytic reaction media, including time, temperature, pH, and enzyme/substrate ratio, optimizing for maximum activity of the enzyme (Table 1). Lee et al. [45] used eight proteases to hydrolyze duck processing by-product to produce antioxidant peptides (Table 2). Based on the hydroxyl radical scavenging activity of various enzymatic extracts, pepsin was selected to produce antioxidant peptides. The enzymes produce a mixture of peptides with a different degree of hydrolysis (DH) which also could be responsible for the different range of antioxidant capacity. This was evidenced by Li et al. who utilized three enzymes and one cocktail to incubate with porcine collagen, with a long duration, and assessed the antioxidant activities and degree of hydrolysis [43]. Among the four hydrolysates, the cocktail hydrolysate exhibited the highest radical scavenging activity (87.18%) and possessed the highest DH (55.32%). Furthermore, DH of the hydrolysates increased with reaction time and the metal chelating activity also increased with a high value of DH. The report of Liu et al. validated the antioxidant activity of porcine blood plasma protein hydrolysate, indicated by thiobarbituric acid-reactive substance (TBARS) values in a liposome-oxidizing system [38]. Thus, the categories of enzymes and degree of hydrolysis could be combined effects on the antioxidant activities of hydrolysate and peptides.

### 2.2. Approaches for Measuring Antioxidant Capacity 

**Figure 1:**
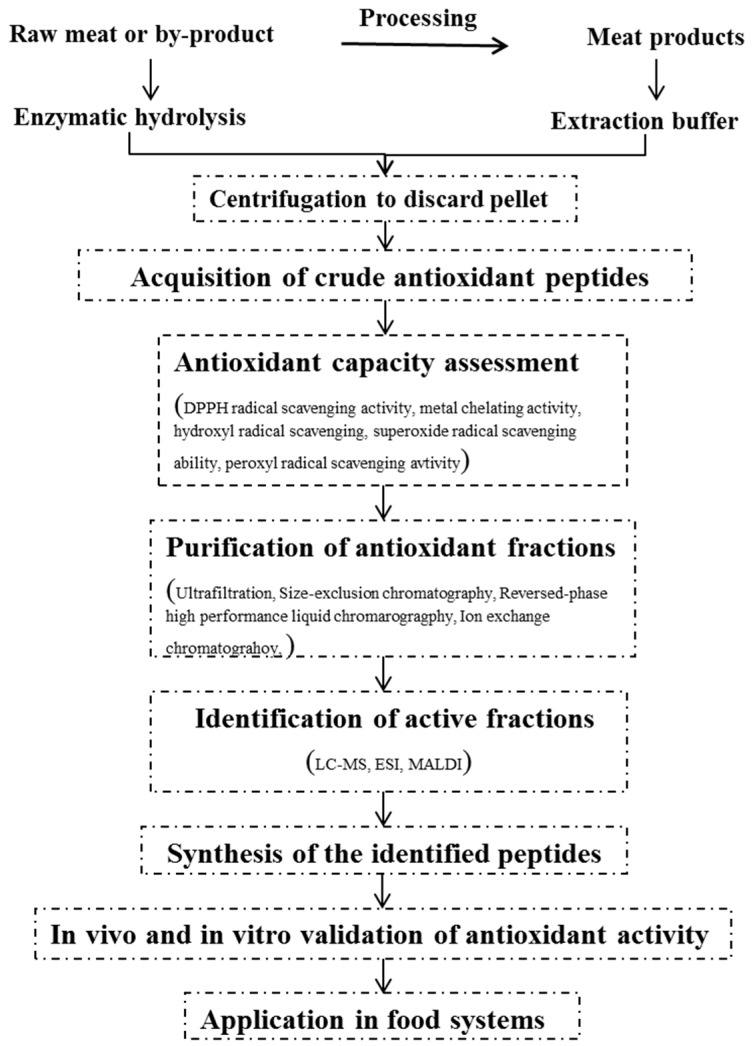
Schematic diagram for the production of antioxidant peptides from meat proteins.

Measuring the antioxidant capacity is an essential process in validating the functional property of enzymatic hydrolysates or crude solvent extracts in order to determine which fractions of the purification step would be subject to purification using mass spectrometry analysis and identification of amino acid sequence of peptides (Figure 1). Methods have been developed to test the antioxidant activity of food compounds and biological samples over decades that have been comprehensively reviewed in several papers [46,47,48]. Up to date, no specific method has been sufficient to characterize the overall antioxidative potential of protein hydrolysate, partially purified peptides, and individual peptides. Therefore, more than two detection assays are commonly used for measuring non-peptidic antioxidants to comprehensively evaluate the antioxidant activity. The methods used for assessing the antioxidant properties of peptides derived from meat proteins are listed in Table 1. Basically, the methods can be broadly divided into in vivo and in vitro assays [13]. Though there is a great deal of evidence that in vitro assays can detect high antioxidant activities in purified peptides [21,22,23,37], whether this functions in peptides in the human body can be challenged due to the barriers of degradation and modification by the intestine, vascular system, and liver [49]. Thus, in vivo assays including animal studies and clinical trials should be further conducted to confirm the bioavailability and functionality of antioxidant peptides. 

#### 2.2.1. Chemical Reactions

Generally, based on the chemical reaction involved, the aim of these assays is to relatively quantify the hydrogen atom donating, electron transferring, or metal ion chelating ability of the target substrate [46]. The majority of assays based on hydrogen atom donating include the oxygen radical absorbance capacity (ORAC), and the total radical trapping antioxidant parameter (TRAP). Those methods are widely applied for assessing the antioxidant activity of phenolic compounds and other phytochemicals, while few have been used for analyzing peptides in this context. The electron transferring based assays are to measure the reduced power of the oxidants, setting color changes as indicators and which contain the trolox equivalent antioxidant capacity (TEAC), the ferric ion reducing power (FRAP), and the DPPH radical scavenging capacity. These kinds of assays constitute a large portion of the relevant studies (Table 1) giving the ability to compare the antioxidant capacity of peptides or hydrolysate from different sources. For example, the peptide DLEE isolated from Xuanwei ham exhibited higher DPPH radical scavenging capacity of 74.45% at 0.5 mg/mL than that of peptide SAGNPN derived from Spanish dry-cured ham with 50% DPPH radical scavenging capacity at 1.5 mg/mL [19,22]. Wang et al. identified seven antioxidant peptides from hydrolysates of duck breast meat. The peptide LQAEVEELRAALE derived from myosin heavy chain showed higher DPPH radical scavenging capacity than that of peptide IEDPFDQDDWGAWKK derived from alpha-enolase [37]. Ferrous ion chelating assay has been regarded as an efficient tool to measure the antioxidant capacity of peptides and this was applied in the study by Li et al. who purified the peptide QGAR from a cocktail mixture of hydrolysate of porcine skin collagen [43]. 

Apart from the aforementioned methods, the ability of peptides to scavenge the individual ROS is also an alternative to evaluating the antioxidant capacity. This category of methods includes the superoxide anion (O_2_•^−^), hydroxyl (•OH), peroxyl (ROO•), and alkyl radical scavenge capacity and is widely employed by researchers [29,33,39,45,50]. Electron spin resonance (ESR) spectrometry acts as an available technique to measure the radical-scavenaging activity of antioxidants against free radicals including DPPH, O_2_•^−^, and ROO•. The principle of ESR spectroscopy is that the electron subjected to an applied magnetic field causes the energy to decrease or increase correlated parallel or antiparalled to the direction of the magnetic field [51]. Antoxidant peptides are reported to retard lipid oxidation and prevent foods from deterioration and discoloration [46]. So, the indicators reflecting the extent of lipid oxidation could be used as parameters to evaluate the antioxidant capacity of peptides. The TBARS, peroxide value (PV) and inhibition of linoleic acid autoxidation are all implied for lipid oxidation and have been applied in antioxidant capacity measurement of enzymatic hydrolysate of bovine skin gelatin [42], porcine plasma [39], and porcine skin collagen [43]. 

#### 2.2.2. Cell Model

In vitro, various cell culture models are valuable tools to assess the bioavailability of antioxidant peptides, as they take advantage of rapid and inexpensive screening compared to animal models and human clinical trials. In addition, the cultured cell model could also give more biologically relevant information about cell uptake, distribution, and metabolism of antioxidant compounds. Kim et al. employed rat liver cells (Ac2F) to subject to oxidant stress by incubating with 1 mM tert-butyl hydroperoxide (t-BHP) in the presence and the absence of purified peptides [42]. MTT assays revealed that t-BHP caused a decrease of cell viability by 48% while pre-incubation of the cell with fraction PII (0.5 mg/mL) increased cell viability by approximately 30% suggesting that PII can protect Ac2F from lipid peroxidation induced by t-BHP. Lee et al. studied the protective effects of peptide HTVGCMPG purified from duck skin hydrolysate on 3.5% alcohol-induced damaged normal liver cells (Chang cell, ATCC CCL-13) [29]. Detection on fluorescent DCF and morphology of the nucleus revealed that the antioxidant peptide inhibited the production of ROS and cell death against alcohol-induced liver cell damage. This evidence further demonstrates that the mechanism of the action of antioxidants goes beyond the antioxidant activity of the scavenging free radical [52], and also includes the regulation of apoptosis-related pathway in cell environments [29]. 

#### 2.2.3. Animal Model

The positive effect of antioxidant peptide on human health promotion concerns the following factors: intestinal digestion of peptide, absorbed into the intestinal lymphatic system and finally transported to the target sites [53]. The animal model is an efficient strategy to evaluate the bioavailability of antioxidant peptides in vivo. However, only a limited number of studies have been conducted to assess the biological potential of protein hydrolysate or isolated peptide derived from meat proteins in this context using animal models. In the animal model, biomarkers of lipid and protein peroxidation are the main indicators used to monitor changes in oxidative stress in vivo [54]. Additionally, SOD, CAT, and GSH-Px are responsible and useful for scavenging superoxide radical and hydroxyl radicals in biological systems such that they have become the important parameters to be applied in animal models [55]. Lee et al. used Wistar rats to investigate the effect of oral administration of gelatin hydrolysate on alleviating ethanol-induced oxidative stress [45]. In this case, antioxidative enzyme activities of SOD, CAT, and GSH-Px were increased significantly in the presence of gelatin hydrolysate compared to the control group. Sun et al. reported that ICR mice were used as an aging animal model to be treated with d-galactose to determine in vivo antioxidant ability of chicken protein hydrolysate [31]. Peptides significantly improved the antioxidative enzyme activities of SOD, CAT, and GSH-Px and decreased the MDA level compared to the d-galactose induced aging mice group thus indicating the antioxidant properties of purified peptides. 

### 2.3. Purification and Identification of Antioxidant Peptides

Protein hydrolysates or crude peptide extracts possess antioxidant capacity to a limited extent and contain the various sizes of peptides and amino acid composition with either inactive or active activity. In order to get to know the structural characteristics of peptide mixtures and further increase the antioxidant capacity of end-products for application and commercialization, it is essential to isolate single bioactive peptides, which thus involves the purification and identification steps (Figure 1). The techniques of purification are strictly associated with the physical and chemical properties of the peptides. Different molecular weights of peptides could be separated by size-exclusion chromatography (for example, Sephadex G-25, G-35, G-75), an ultrafiltration membrane system using different cut-off sizes (10, 5, 3, 1 kDa) or tangential flow filtration [33,39,43,56]. Ion exchange chromatography was utilized to fractionate different amounts of positive and negative charged peptides [42,44]. Peptides with hydrophilic and hydrophobic properties were separated by reversed-phase high performance liquid chromatography (RP-HPLC) [33,42,45]. To obtain a highly purified fraction or peptide, combined purification methods should be operated sequentially. Generally, the fraction with the highest antioxidant capacity is used to identify the amino acid sequence of the peptide. Liquid chromatography followed by tandem mass spectrometry detection (LC-MS/MS) is mostly commonly used to identify the peptide sequence [57]. Matrix-assisted laser desorption/ionization time of fight (MALDI-TOF) and Electron Spray Ionization (ESI) mass spectrometry also are frequently used in identifying the peptide sequence of purified fractions. 

We have used the above mentioned methods and successfully isolated a bioactive peptide with high antioxidant capacity from Xuanwei ham [22]. A crude extract with 85% peptide content was obtained exhibiting 35.34% DPPH scavenging capacity and 78.05% superoxide radical scavenging capacity at a concentration of 1 mg/mL. A size exclusion chromatography column packed with G-25 was used to fractionate four parts from the crude extract and the third part (C) presented the highest scavenging rate of 35.06% on OH• and 36.12% on DPPH radical. Fraction C was further subjected to anion exchange chromatography and divided into a further five peaks. The fifth peak (C5) of the peptide possessed the highest DPPH scavenging capacity up to 82.14% and OH• scavenging rate of up to 54.95% at a concentration of 1 mg/mL. The C5 fraction was then subjected to the last purification step of RP-HPLC and generated seven portions. The seventh portion of C5 with the highest scavenging effect on DPPH free radical was in turn sent to LC-MS/MS to analyze the peptide sequence. Finally, the peptide sequence of DLEE was identified and possessed 74.45% DPPH scavenging ability at a concentration of 0.5 mg/mL [39]. 

## 3. Characteristics of Antioxidant Peptides 

It is of great importance to extract all available information on the structure-activity relationship of antioxidative peptides derived from meat proteins, to provide guides and predictions for evaluating bioactive peptides from certain proteins based on the amino acid sequence and the cleavage bonds of the enzymes. Though the exact mechanism of the antioxidant activity of peptides has not yet been fully understood, several common characteristics of peptides that possess considerable antioxidant capacity have emerged. The peptide sequences and corresponding molecular weights derived from animal proteins and by-products are summarized in Table 1. 

### 3.1. Molecular Weight 

The common feature of the identified peptides shows a size of 4–16 amino acids, and a molecular weight of approximately 400–2000 Da (Table 1). Escudero et al. identified 51 peptides generated from simulated gastrointestinal pork meat by sequential action of pepsin and pancreatin with sizes ranging from 6 to 16 amino acids and molecular weights of 600–1500 Da [58]. Similarly, ninety three peptides were characterized from a size-exclusion chromatography fraction with sizes of 5–20 amino acids and molecular weights of 400–2500 Da and some of which with smaller molecular weights possessed high antioxidant capacity [20]. Lee et al. reported that three fractions were obtained from the hydrolysate of duck processing by-product by ultrafiltration membrane treatment at a range of molecular cutoffs (MWCO) of 30, 10, and 5 kDa using a TFF system [45]. The lower molecular weight of the <5 kDa fraction possessed the highest hydroxyl radical scavenging activity, exhibiting an IC_50_ value of 0.532 mg/mL. Wang et al. separated the porcine plasma albumin and globulin hydrolysate by Alcalase into five groups (<3 kDa, 3–6 kDa, 6–10 kDa, 10–30 kDa, >30 kDa) according to the molecular weight by sequential ultrafiltration and assessed their antioxidant capacity [39]. They concluded that fractions with a small molecular weight (<3 kDa) from two proteins possessed better antioxidant potential with evidence of higher reducing power and scavenging capacity on DPPH, hydroxyl, and superoxide in the <3 kDa fraction compared to large molecular fractions. Liu et al. also reported that the fraction with <3 kDa molecular weight of 17.6% of the plasma protein hydrolysate exhibited higher reducing power and DPPH scavenging activity compared to other fractions with large molecular weight [38]. Finally, the amino acid sequence of the peptide with high antioxidant capacity was identified as HNGN with a molecular weight of 441 Da (Table 1). It can be concluded that smaller molecular weights of crude peptides tend to have higher antioxidant activity which is consistent with previous reports on peptides derived from other sources [59,60,61]. For example, Li et al. revealed that peptides derived from corn gluten hydrolysate with molecular weights of 500–1500 Da had higher antioxidant activity compared to those of peptides above 1500 Da [62]. The fraction of <3 kDa molecular weight also exhibited the highest antioxidant capacity in egg white protein hydrolysate with DPPH scavenging activity of 78.74% at a concentration of 5 mg/mL [63]. 

The molecular weight also affects the routes by which bioactive peptides transfer into target sites [64]. Small molecular weight peptides could easily get through the gastrointestinal barrier and enter peripheral blood to promote its bioavailability at tissue level [65]. Peptides with a size of 2–6 amino acids could be more easily absorbed compared to proteins and single amino acids [66]. In addition, the bioactive peptides should have the ability to undergo further digestion by gastrointestinal proteases which could decrease the peptides and increase the antioxidant capacity in vivo [67]. It was reported that the peptides of rich proline residues could be made more resistant to further degradation by digestive enzymes [58,68]. 

### 3.2. Constitution and Sequence of Amino Acid 

It is noteworthy that free amino acids could also exhibit antioxidant capacity in the decreasing order of Trp, Tyr, Met, followed by Cys, His, Phe with no antioxidant property in the remaining amino acids [50]. However, they have not been found to be effective as antioxidants in food and biological systems. On the other hand, some of these free amino acids which have little or no effect individually could exhibit higher antioxidant capacity in peptides [69,70]. Thus, the amino acid sequence plays a vital role in determining the antioxidant activity of the peptides. All the amino acids could be classified into the following categories based on their chemical and physical properties: aromatic amino acids, hydrophobic amino acids, acidic and basic amino acids, and cysteine [71]. 

The category of aromatic amino acids contains tyrosine (Tyr), histidine (His), tryptophan (Trp), and phenylalanine (Phe). The amino acids can donate protons to the electron deficient radicals contributing to the radical-scavenging properties of the amino acid residue [72]. The His-containing peptides which exert high antioxidant capacity have been mainly investigated [73,74]. This may be explained by the imidazole ring in the R group having hydrogen donating ability, lipid peroxyl radical trapping, and metal ion-chelating ability [75]. The peptides of HNGN derived from porcine plasma hydrolysate [38] and HTVGCMPG isolated from duck skin hydrolysate [29] contain the same His in the *N*-terminus of the sequence, with strong scavenging ability on DPPH, OH^−^, and superoxide. These also occur in the peptide of HFGDPFH purified from fermented marine blue mussel [72] and HGPLGPL derived from Hoki skin gelatin protein hydrolysate [76], suggesting that the position of His in the *N*-terminus could contribute to the antioxidant capacity. In order to further observe the effect of His-containing peptide on peptide bioactivity, Chen et al. designed a total of 28 synthetic dipeptides, tripeptides, and tetrapeptides based on a pentapeptide of LLPHH isolated from soybean protein hydrolysate [77]. In the same context, 114 tripeptides were created containing either His or Tyr residue to evaluate their antioxidant activity using a linoleic acid peroxidation system [30]. The amino sequence of Pro-His-His exhibited higher antioxidant capacity in the linoleic acid system than that of other synthetic peptides, thus presenting evidence that the specific amino acid sequence of peptides is important for their properties. This was also validated by the oxidation of NRVYIHPF mediated by copper (II)/ascorbate in that the *N*-terminal NRVY sequence had a predominant effect on determining the reactivity of His residue compared to the remaining tetrapeptides of IHPF [78]. In addition, tripeptides with Trp and Tyr residue at the C-terminus could exert strong radical scavenging activities [30]. 

Hydrophobic amino acids include alanine (Ala), isoleucine (IIe), leucine (Leu), proline (Pro), Phe, Tyr, and Trp. Despite the priority of Phe, Tyr, and Trp as hydrogen donors, the hydrophobic amino acids could increase the presence of peptides at the water-lipid interface and then access to scavenge free radicals from the lipid phase [79]. Ohata et al. identified a peptide of GYP, which contained two continuous hydrophobic amino acids, Tyr and Pro. They found that this peptide showed an extremely high hydroxyl radical scavenging activity (97.6%) at 100 mM concentration [26]. More than half of the peptide sequences derived from animal proteins with strong scavenging radical capacity contain hydrophobic amino acids such as Ala in GLAGA [19] and AVCGAAVAGT [45], IIe in IEAEGE [30], as well as Leu in DLEE [22] and DLYA, SLYA [34]. However, it is not an infallible rule that peptides with hydrophobic amino acids have potential antioxidant activity. For example, Tsuge et al. reported that the dipeptide of Ala-His designated from Ala-His-Lys had no antioxidant activity [80]. 

There are two acidic amino acids of aspartic acid (Asp), glutamic acid (Glu) and three basic amino acids of arginine (Arg), lysine (Lys), and His. They utilize carbonyl and amino groups in the side chain which function as chelators of metal ions [81]. We purified a peptide with an amino sequence of DLEE containing three acidic amino acids residues and one hydrophobic amino acid residue and it possessed high DPPH scavenging ability [22]. The peptide sequence of QGAR with Arg at the C-terminus which was isolated from porcine collagen hydrolysate, exhibited ferrous ion chelating activity [43]. Additionally, cysteine also contributed to antioxidant potential because the sulfydryl in the R groups could act as radical scavenger with reducing power [82]. In the aforementioned synthetic peptides, Cys-containing tripeptides showed strong scavenging ability on peroxynitrite [30]. 

## 4. Implication 

The main benefit of developing an innovative and safe antioxidant peptide is to use to protect the human body from damage of oxidative stress as well as the risk of various degenerative diseases. The commercialized uses of antioxidant peptides are spread over the fields of functional foods, nutraceuticals, and cosmeceuticals. There is already a range of antioxidant peptides which is allowed to be incorporated into foods in the form of additives in some countries such as Japan and USA. However, popularization of antioxidant peptides derived from animal proteins still has a long way to go. It has been confirmed that identified peptides can exhibit strong antioxidant capacity in vitro. The lack of animal model experiments and human clinical trials has limited the progress in testing the bioavailability and then application in the consumer market. Thus, further research should be conducted to investigate the potential effects of bioactive peptides in vivo. In addition, the structure-activity relationship of peptides has not been fully established and little is known at present of the structural information of antioxidant peptides from the various protein sources. 

## Figures and Tables

**Table 1:** Antioxidant hydrolysate and peptides generated from meat proteins and by-products.

Source	Antioxidant Fractions	Preparation	Parental Protein	Antioxidant Activity Assessment	Mw(Da)	IC_50_ or Scavenging Activity	Reference
Fermented meat sauce	GYP	Centrifugation of fermented mash	-	DPPH radical-scavenging activity Hydroxyl radical scavenging	406.26	OH^−^ (97.6% at 100 mM)	[26]
Pork hams	Water-soluble and salt-soluble protein hydrolysate	Pepsin in solution pH 2.0 at 37 °C for 2–10 h 1/100 (w/w)	-	Linoleic acid emulsion system Ferric-reducing antioxidant power DPPH radical scavenging activity	<7 kDa	DPPH (50% at 0.1% (w/w))	[36]
Spanish dry-cured ham	Elution zone between 220 and 345 mL	0.01 N HCl extraction buffer		DPPH radical-scavenging activity Superoxide ion extinguishing ability	1700	DPPH (fraction 220–345 mL, 39%–92%) O^2−^ (fraction 240–280 mL with 41.67%, 50.27%)	[18]
Spanish dry-cured ham	SAGNPN, GLAGA (2 most active peptides from 27 identified peptides)	0.01 N HCl extraction buffer	Integrin alpha-3, Collagen, type VII, alpha 1	DPPH radical-scavenging activity, Ferric-reducing antioxidant power	558.24 387.21	DPPH (SAGNPN, 50% at 1.5 mg/mL), Reducing power (GLAGA, 0.5 units of absorbance, at 1 mg/mL)	[19]
Spanish dry-cured ham	SNAAC (the most active peptide from 93 identified peptides)	0.01 N HCl extraction buffer	myosin heavy chain 1,4	DPPH radical-scavenging activity, Ferric-reducing antioxidant power	464.17	DPPH (IC_50_ = 75.2 µM) Reducing power (IC_50_ = 205 µM)	[20]
Jinhua ham	GKFNV	0.01 N HCl extraction buffer	-	Hydroxyl radical scavenging activity, DPPH radical scavenging activity, Fe^2+^ chelating ability	564.4	Crude extracted peptides: OH^−^ (54% at 1 mg/mL) DPPH(77.39% at 1 mg/mL) Fe^2+^ (63.20% at 1 mg/mL)	[21]
Xuanwei ham	DLEE	0.2 mM phosphate buffer (pH 7.2)	-	DPPH radical-scavenging activity Hydroxyl radical scavenging activity Superoxide ion scavenging activity	505.2	DPPH(74.45% at 0.5 mg/mL)	[22]
Duck breast meat	LQAEVEELRAALE IEDPFDQDDWGAWKK	Protamex, 0.75/100 (w/w) 50 °C for 4 h, pH 6.0	Myosin heavy chain Alpha-enolase	Hydroxyl radical scavenging activity DPPH radical scavenging activity, Fe^2+^ chelating ability	1471.8 and 1851.9	LQAEVEELRAALE: DPPH (93.36% at 1 mg/mL), Fe^2+ ^(87.13% at 1 mg/mL). IEDPFDQDDWGAWKK: OH^−^ (46.51% at 1 mg/mL)	[37]
Duck skin	HTVGCMPG	Pepsin in Glycine-HCl pH 2.0 at 37 °C for 8 h 0.2/1000 (w/w)	-	Hydroxyl radical scavenging activity DPPH radical scavenging activity Alkyl radical scavenging activity Superoxide radical scavenging activity	941.43	OH^−^ (IC_50_ = 32.6 μg/mL) DPPH (IC_50_ = 22.7 μg/mL) Alkyl (IC_50_ = 55.1 μg/mL) O^2−^(IC_50_ = 49.8 μg/mL)	[29]
Duck skin	AVCGAAVAGT	Pepsin in Glycine-HCl pH 2.0 at 37 °C for 8 h 0.2/1000 (w/w)	-	Hydroxyl radical scavenging activity	1096	OH^−^ (IC_50_ = 75 μg/mL )	[45]
Gelatin from duck skin	gelatin hydrolysate of two enzymes combination	Collagenase pH 7.0, 37 °C , 8 h and pepsin, pH 2.0, 37 °C, 8 h with 0.2/1000 (w/w)	-	Hydroxyl radical scavenging activity DPPH radical scavenging activity Alkyl radical scavenging activity	-	DPPH (IC_50_ = 0.632 mg/mL) OH^−^ (IC_50_ = 0.222 mg/mL) Alkyl (IC_50_ = 0.708 mg/mL)	[41]
Porcine myofibrillar protein	DSGVT IEAEGE DAQEKLE EELDNALN VPSIDDQEELM	Papain in water pH 7.0, at 37 °C for 24 h with 1/100 (w/w)	Actin Tropomyosin Myosin heavy chain	Measurement of hydroperoxides in a Peroxidation System DPPH radical scavenging activity Metal ion chelating activity	650.3, 646.4 832.5, 916.9 1275.0	-	[30]
Chicken breast protein	Breast protein hydrolysate	Papain in water for 6.15 h at 51.2 °C, pH 6.5 1.5/1000 (w/w)	-	Reducing power assay DPPH scavenging activity assay ICR mice model	-	Reducing power (0.5 at 2.37 mg/mL) DPPH (IC_50_ = 1.28 mg/mL)	[31]
Bovine brisket sarcoplasmic proteins	Potential peptides of Sarcoplasmic protein hydrolysates: EAWAEDVDLRVN GGWQMEEADDWLR GWQMEEADDWLR RIGEEYIADLDQLRKLL VFEWEAFAR AIMENANVLAR LAIMENANVLAR	Papain in water for 24 h at 37 °C, pH 7.0 with 1:100 (w/v).	Pyruvate kinase, Phosphorylase Phosphoglycerate kinase 1 Fructase-biphosphate aldolase	DPPH scavenging activity assay Fe^2+^ chelating ability assay Ferric ion reducing antioxidant power (FRAP)	Ranging from 1154.56 to 2045.13	DPPH(18.68% at 1 mg/mL) in NUFH FRAP (6.85% µg/5 mg peptide) in10-kDa-UFH Fe^2+^ (82.42% at 5 mg/mL) in 3-kDa-UFH (show the most active fractions)	[32]
Porcine blood plasma	HNGN	Alcalase at 55, pH 8.0 for 5 h with 2:100 (g/g)		Thiobarbituric acid-reactive substances (TBARS) DPPH scavenging activity assay Ferric ion reducing antioxidant power (FRAP) Metal chelating activity	441	FRAP (236.4 µM at 1 mg/mL)	[38]
Venison muscle	MQIFVKTLTG DLSDGEQGVL	Papain in PB at 37 °C for 8 h pH 6.0 with 1/2500 (w/w)	-	Hydroxyl radical scavenging activity DPPH radical scavenging activity Superoxide radical scavenging activity Peroxyl radical scavenging activity	1137.40 1023.07 (predicted)	DPPH (IC_50_ = 77 µg/ml) OH^−^ (IC_50_ = 44 µg/ml) O_2_^−^ (IC_50_ = 217 µg/ml) Peroxyl (IC_50_ = 85 µg/ml)	[33]
Chicken breast meat	Hydrophilic fraction	Water extraction	-	ABTS radical cation decoloration assay	-	TEAC (total fractions 2.4 µmol/g)	[24]
Porcine muscle	DLYA, SLYA,VW	Papain	Actomyosin	-	-	-	[34]
Porcine skin collagen	QGAR	Cocktail mixture of three enzymes: PP, PS, PB at 25 °C pH 7.5 for 24 h with 1/125 (w/w)	Collagen	Linoleic acid oxidation system Ferrous ion chelating assay DPPH radical scavenging assay	430.2	DPPH (37.27% at 20 mM) Linoleic acid (38.48% at 20 mM)	[43]
Porcine plasma	Hydrolysates	Alcalase at 55 °C pH 7.5 for 24 h with 1/1000 (w/w)	Albumin and globulin	Reducing power DPPH radical scavenging activity Inhibition ability of lipid peroxidation Hydroxyl radical scavenging activity Superoxide radical scavenging activity	-	-	[39]
Porcine blood	Pepsin hydrolysate Papain hydrolysate	Pepsin at 37 °C pH 2 for 5 h with 1/25 (w/w) Papain at 37 °C pH 8 for 16 h with 1/20 (w/w)	Plasma proteins	Antioxidant activity in linoleic acid system DPPH radical-scavenging activity Iron(II)-chelating activity	-	DPPH (48.4% and 43.1% at 500 µg/ml for PPE and PPA respectively)	[35]
Porcine blood	Porcine hydrolysates	2% Alcalase for 4 h pH 8.5 and followed by 1% Flavourzyme for 6 h pH 7.5, at 50 °C for 6 h	Hemoglobin	Reducing power Ferrous ion chelating ability DPPH radical-scavenging activity	-	Fe^2+^ (63.54% at 5.0 mg/mL)	[40]
Bovine skin gelatin bovine	GE-Hrp-GP-Hrp-GA-Hrp GP-Hrp-GP-Hrp-GP-Hrp-G GP-Hrp-GP-Hrp-GP-Hrp	Alcalase, pronase E amd collagenase sequentially	Collagen	TBARS	826 877 820	-	[42]

**Table 2:** Optimum conditions for the hydrolysis from duck processing by-product and hydroxyl radical scavenging activity of various enzymatic extracts (mg/mL).

Enzymatic Hydrolysate	Buffer	pH	Temperature (°C)	Time (h)	Hydroxyl Radical Scavenging Activity (%)
a-Chymotrypsin	Phosphate	7.0	37	8	19.45 ± 0.41
Alcalase	Phosphate	7.0	50	8	26.61 ± 0.56
Flavozyme	Phosphate	7.0	50	8	29.46 ± 0.39
Neutrase	Phosphate	7.0	50	8	24.45 ± 0.27
Papain	Phosphate	6.0	37	8	34.38 ± 0.32
Pepsin	Glycine-HCl	2.0	37	8	54.29 ± 0.14
Protamax	Phosphate	7.0	50	8	27.74 ± 0.25
Trypsin	Phosphate	7.0	37	8	28.33 ± 0.03

Adapted from Lee et al. [45].

## Abbreviations

The following abbreviations are used in this manuscript:

ROS	reactive oxygen species
RNS	reactive nitrogen species
SOD	superoxide dismutase
BHA	butyl hydroxy anisd
BHT	butylated hydroxytoluene
DH	degree of hydrolysis
TBARS	thiobarbituric acid-reactive substance
ORAC	oxygen radical absorbance capacity
TRAP	total radical trapping antioxidant parameter
TEAC	trolox equivalent antioxidant capacity
FRAP	ferric ion reducing power
O_2_•^−^	superoxide anion
•OH	hydroxyl
HO_2_•	hydroperoxyl
ROO•	peroxyl
ESR	electron spin resonance
PV	peroxide value
RP-HPLC	reversed-phase high performance liquid chromatography
